# Cost-Effectiveness of Primary HPV Screening Strategies and Triage With Cytology or Dual Stain for Cervical Cancer

**DOI:** 10.1177/1073274820922540

**Published:** 2020-05-06

**Authors:** Tanitra Tantitamit, Nipon Khemapech, Piyalamporn Havanond, Wichai Termrungruanglert

**Affiliations:** 1 Department of Obstetrics and Gynecology, Faculty of Medicine, Srinakharinwirot University, Nakhon Nayok, Thailand; 2 Department of Obstetrics and Gynecology, Faculty of Medicine, Chulalongkorn University, and King Chulalongkorn Memorial Hospital, Bangkok, Thailand

**Keywords:** cervical cancer, cost-effectiveness analysis, biomarkers, cancer screening, human papillomavirus DNA tests

## Abstract

To identify the optimal cost-effective strategy for cervical cancer screening program in Thailand by comparing the different algorithms which based on the use of primary human papilloma virus (HPV) assay. We use a Microsoft Excel–based spreadsheet to calculate the accumulated cases of preinvasive and invasive cervical cancer and the budget impact of each screening program. The model was developed to determine the cost-effectiveness of 3 screening strategies: pooled HPV test with reflex liquid-based cytology triage, HPV genotyping with reflex p16/ki67 dual stain cytology, and pooled HPV test with dual stain. The main outcomes were the total cost, incremental quality-adjusted life years (QALYs) and incremental cost-effectiveness ratios (ICERs). Strategy entailing primary HPV genotyping and reflex dual stain cytology is the least costly strategy (total cost US$37 893 407) and provides the similar QALY gained compared to pooled high-risk HPV testing with reflex dual stain (Average QALY 24.03). Pooled HPV test with reflex dual staining is more costly compared to strategy without reflex dual staining. The ICER was US$353.40 per QALY gained. One-way sensitivity analysis showed that the model is sensitive to the cost of dual stain and the cost of cancer treatment. Decreasing the incidence of cervical cancer case and increasing the QALYs can be successful by using dual stain cytology as the triage test for pooled HPV test or HPV genotyping. The result of our analysis favors the use of HPV genotyping with the reflex dual stain as it offers the most QALY at the lowest cost.

## Introduction

Cervical cancer is the fourth most common cancer in women of about 570 000 of new cases in 2018.^1^ Most of the cases are distributed in developing countries.^1^ Ineffective screening program and low level of coverage of the target population are the main reasons of a high burden of disease.^2–4^ In Thailand, the current cervical cancer screening program has been found to be ineffective.^5^ The main strategy used is cytology-based screening. Human papilloma virus (HPV) testing has been used in conjunction with cytology and used for triage of cervical cytology showing atypical squamous cells of undetermined significance (ASCUS). To improve the efficacy and increase the coverage of screening, several new strategies have been studied.^6–11^ Our previous study compared the cost and benefit of 4 different cervical cancer screening strategies involving primary HPV 16/18 genotyping, high-risk HPV testing, liquid-based cytology, and conventional cytology.^12^ Model predictions indicated that the most cost-effectiveness strategy is primary high-risk HPV testing by reducing cost and also increase the detection of cervical intraepithelial neoplasia 2 and 3. However, the primary HPV testing still has some problem because of its high sensitivity and low specificity. This may cause overtreatment. The concept of dual stain has been introduced to decreased overtreatment case. One systematic literature review showed that across all age groups in a screening population, dual staining was significantly more sensitive than and equally specific as cytology.^13^ Specificity gains resulted in fewer false positives and an increase in the number of correct referrals to colposcopy. Dual staining with p16/Ki-67 cytology is an attractive biomarker approach for triage in cervical cancer screening.^13^

Our recent study assessed the clinical and cost-effectiveness of HPV primary screening triage with p16/Ki-67 dual stain cytology compared to cytology. The preliminary result suggests that screening by use of HPV genotyping test and dual stain cytology as the triage test for other high-risk HPV positive women as a primary screening test in Thai population 30 to 65 years old is expected to be more cost-effective than cytology.^14^ Based on the available results of cost-effectiveness analysis study in Thailand, the objective of this study is to evaluate the cost and effectiveness of HPV-based primary screening strategies with different triage including Papanicolaou (Pap) cytology and p16/Ki67 dual stain cytology in Thai population 30 to 65 years old in order to implement the most appropriate strategy to our country.

## Materials and Methods

### Epidemiologic Modeling

**Figure 1.**
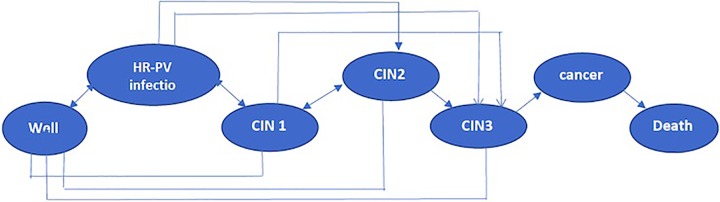
Model natural history of cervical cancer.

We developed a computer simulation model (A Microsoft Excel–based spreadsheet) to calculate the number of accumulated cases of cervical intraepithelial neoplasia, invasive cervical cancer, and budget impact of each screening program. The model of natural history was constructed (Figure 1). Individual women enter the model at age of 30 years. They face the yearly probabilities of transitioning between HPV-related health stages, including well, high-risk HPV infection, cervical intraepithelial neoplasia, invasive cervical cancer, and death. The simulation model continued until women had died or diagnosed with cervical intraepithelial neoplasia 2, cervical intraepithelial neoplasia 3, and cancer.

### Screening and Management Algorithms

The study population for our simulation was a closed cohort representing the Thai population 30 to 65 years of age which is an approximately 26.6% of the total population. The screening coverage was 50% of the target population.^15,16^ Based on the total number of Thai population (in 2015) and excluding the women with HIV infection, history of hysterectomy and pregnant, 7 953 963 were eligible for screening.^17^

**Figure 2.**
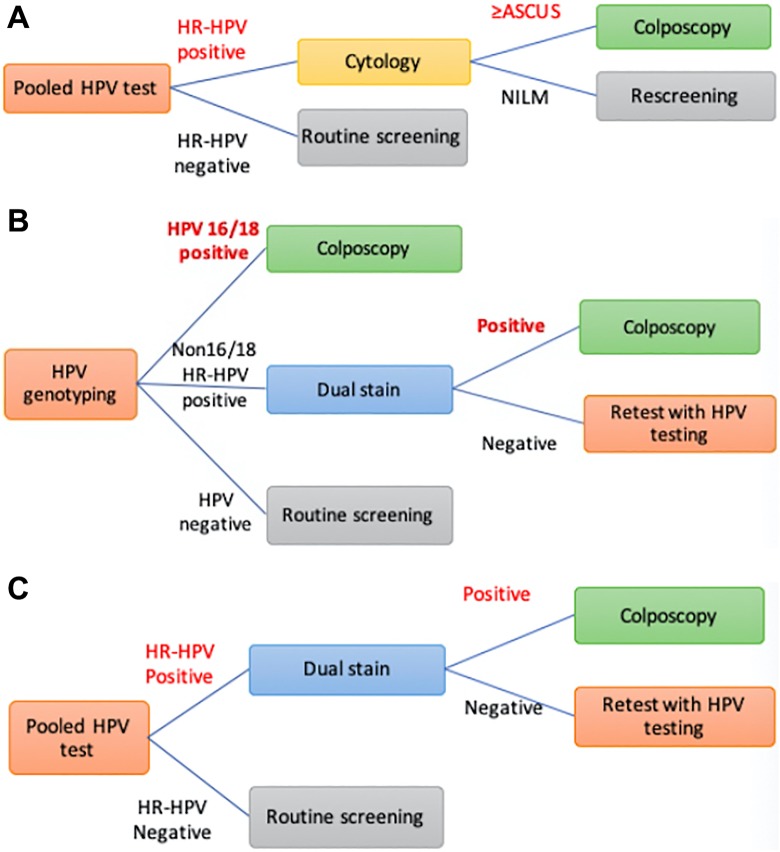
Screening model. A, Pooled HPV test with reflex LBC triage. B, HPV genotyping test with reflex dual stain. HPV indicates human papilloma virus.

We examined 3 different algorithms of primary HPV assay based on the evidence of cost-effectiveness data from our previous studies.^12,14^ According to the clinical guideline of the National cancer institute of Thailand, all strategies were considered a 5-year interval. The decision tree models of screening and management algorithms are displayed in Figure 2.

1. High-risk HPV testing with reflex liquid-based cytology (LBC) triage: Pooled high-risk HPV testing is a primary tool, followed by LBC for women with the high-risk HPV positive result. A cytology of ASCUS or worse leads to immediate colposcopy. A repeat HPV testing at 12 months will be performed for HR-HPV-positive women with normal cytology. If the result of high-risk HPV is negative, the women will return to routine screening in 5 years (Figure 2A).
2. Human papilloma virus with 16/18 genotyping and reflex dual stain: Screening with HPV genotyping then refers to colposcopy if the result is positive for HPV 16 or 18. The dual staining is performed in cases of other 12 high-risk HPV positive and those with positive result undergo colposcopy. For those who negative dual staining, HPV testing will be done at 12 months interval. Women with a negative result of HPV genotyping return to screening in 5 years (Figure 2B).
3. High-risk HPV testing and reflex dual stain: using high-risk HPV testing alone every 5 years followed by dual stain if the result is positive for high-risk HPV. The women with positive results of both high-risk HPV testing and dual stain will refer to perform colposcopy. Repeat the HPV test in 12 months for a woman with HR-HPV-positive and -negative dual staining (Figure 2C).

### Model Assumptions

Colposcopy was considered to be a gold standard for diagnosis of a precancerous lesion with 100% sensitivity and specificity. Disease progression and regression were assumed to be constant over time and not to be age-specific. The model assumes that the loss to follow-up rate in all strategies were the same and thus will not affect the comparative result. Based on expert’s opinion, we assumed that all cervical intraepithelial neoplasia 3 cases and 50% of cervical intraepithelial neoplasia 2 received treatment. Women with cervical intraepithelial neoplasia 1 or posttreatment cervical intraepithelial neoplasia 2+ returned to follow-up every 6 months. Cervical intraepithelial neoplasia 1 patients with 2 times of negative results and cervical intraepithelial neoplasia 2+ patients with 4 times of negative results would return to routine screening.^18^

Four percent of cervical intraepithelial neoplasia 2 to 3 and 20% of invasive cervical cancer were assumed to be recurred after treatment^19^; they would stay in the current status or progress to more severe states in the model. We considered both deaths from cervical cancer and all-cause mortality.

### The Model Outcome, Cost Data, and Cost-Effectiveness

Based on case values used in the model were mainly based on the data from the published literature referenced in Tables 1 and 2. The outcomes for the model included: screening performances, a total number of cancer and precancerous cases detected, and life expectancy adjusted for quality of life. Age-adjusted annual probabilities of death without cervical cancer were derived from the general population estimates reported in Estimated Generation Life Tables for Thailand of 5-Year Birth Cohorts: 1900-2000.^20^ We conducted the analysis from provider’s perspective. The cost component used for the cost evaluation are depicted in Table 2. Screening costs and treatment cost were derived from our previous studies which based on the cost of the Center of Health Assurance at King Chulalonglongkorn Memorial Hospital and Roche Diagnostic, Thailand.^12,14,21^ Only direct medical cost was included. Indirect costs such as loss of productivity and transportation costs were assumed to have the same among patients. The cancer treatment cost is the median cost across all stages. All clinical and cost inputs were discounted at an annual rate of 3.5%.^22^ The results of cost-effectiveness analysis were presented by incremental cost-effectiveness ratios (ICERs) per quality-adjusted life years (QALY) which is the additional cost divided by additional QALY, compared to the next most costly strategy. We used Thailand’s gross domestic product (GDP) per capita to evaluate the most cost-effectiveness strategy which is suggested by the World Health Organization (WHO). An ICER of less than 3 times the per capita GDP would be considered cost effective.^23^

**Table 1.** Clinical Parameters.

The Performance of Screening Test^26,27^	Input Value
Cytology (threshold = ASCUS)	
Sensitivity of cytology for cervical intraepithelial neoplasia 2	53.20%
Sensitivity of cytology for cervical intraepithelial neoplasia 3	57.70%
Sensitivity of cytology for invasive cervical carcinoma	57.70%
Specificity of cytology	73.40%
HPV testing	
Sensitivity of pooled high-risk HPV testing for cervical intraepithelial neoplasia 2	86.40%
Sensitivity of pooled high-risk HPV testing for cervical intraepithelial neoplasia 3	89.90%
Sensitivity of pooled high-risk HPV testing for invasive cervical carcinoma	89.90%
Specificity of pooled high-risk HPV testing	62.70%
Sensitivity of genotyping 16/18 for cervical intraepithelial neoplasia 2	43.60%
Sensitivity of genotyping 16/18 for cervical intraepithelial neoplasia 3	53.40%
Sensitivity of genotyping 16/18 for invasive cervical cancer	59.20%
Specificity of genotyping 16/18	91.90%
Dual staining	
Pooled HPV triage	
Sensitivity for cervical intraepithelial neoplasia 2	86.80%
Sensitivity for cervical intraepithelial neoplasia 3	89.80%
Specificity for cervical intraepithelial neoplasia 2+	71.40%
Sensitivity for invasive cervical cancer	93.80%
Epidemiology data^26–33^	Input value
Prevalence of high-risk HPV	5.6%
Prevalence of HPV16 and 18	1.7%
Prevalence of cervical intraepithelial neoplasia 1	0.6%
Prevalence of cervical intraepithelial neoplasia 2	0.3%
Prevalence of cervical intraepithelial neoplasia 3	0.8%
Prevalence of invasive cervical cancer	0.075%
General population annual death rate	0.800%
% of high grade squamous intraepithelial lesion (HSIL) + population that is HPV+	88.4%
% of low grade squamous intraepithelial lesion (LSIL) population that is HPV+	61.5%
% of atypical squamous cells of undetermined significant (ASCUS) population that is HPV+	21.4%
% cervical intraepithelial neoplasia 1 that are high-risk HPV 16/18	13.6%
% cervical intraepithelial neoplasia 2 that are high-risk HPV 16/18	23.1%
% cervical intraepithelial neoplasia 3 that are high-risk HPV 16/18	50.3%
% of invasive cervical carcinoma that are high-risk HPV 16/18	75.0%
Natural history parameters ^31,34–42^	Input value
Progression	
Well to high-risk HPV infection	3.20%
Transformation from high-risk HPV (12 types) to:	
cervical intraepithelial neoplasia 1	9.10%
cervical intraepithelial neoplasia 2 (3year FU Luyten)	0.10%
cervical intraepithelial neoplasia 3	0.10%
Transformation from high-risk HPV 16/18 to:	
cervical intraepithelial neoplasia 1	7.30%
cervical intraepithelial neoplasia 2	2.20%
cervical intraepithelial neoplasia 3	2.00%
Progression from cervical intraepithelial neoplasia 1	
to cervical intraepithelial neoplasia 2	3.10%
to cervical intraepithelial neoplasia 3	0.90%
Progression from cervical intraepithelial neoplasia 2	
to cervical intraepithelial neoplasia 3 (2year FU, Luyten)	4.20%
to invasive Cervical Cancer	0.00%
to cervical intraepithelial neoplasia 3 to invasive Cervical Cancer	4.50%
Annual mortality rate for cervical cancer	8.30%
Regression	
Regression from high-risk HPV (12 types) to:	
with NORMAL smear to well	58.60%
with BORDERLINE/MILD smear to well	45.60%
Regression from high-risk HPV 16/18 to:	
with NORMAL smear to well	43.80%
with BORDERLINE/MILD smear to well	21.80%
Regression from cervical intraepithelial neoplasia 1	
to well	21.20%
to high-risk HPV	2.40%
Regression from cervical intraepithelial neoplasia 2	
to well	9.40%
to cervical intraepithelial neoplasia 1	9.40%
Regression from cervical intraepithelial neoplasia 3	
to well	3.80%
to cervical intraepithelial neoplasia 1	1.60%

Abbreviations: HPV, human papilloma virus.

**Table 2.** Cost Parameters.^a^

Cost	Input Value (USD)
Screening costs^21^	
Office visit (routine/repeat screening)	2.00
Cytology test (lab fee)	5.30
Cytology test (professional fee)	3.00
HPV DNA test	17.00
P16/Ki67 dual staining	35.00
Diagnostic costs^21^	
Office visit (diagnostic follow-up)	12.86
Colposcopy plus biopsy	21.42
Treatment costs^21^	
Treatment for cervical intraepithelial neoplasia 2/3	1292.00
Treatment for invasive cervical cancer	7403.00
End of life cancer treatment cost	10019.00
Discounting rate	
Discount rate for cost	0.035
Discount rate for health outcomes	0.035

Abbreviation: HPV, human papilloma virus.

^a^ The currency used was USD (US Dollar exchange rate on March 1, 2019, US$1 = 32 THB).

### Sensitivity Analysis

A one-way sensitivity of cost parameter of the screening tools in each strategy was performed in order to evaluate the uncertainty in which parameters might affect the ratio. The costs were varied 10% below and 3 times above the base case estimation.

## Results

### Base Case Analysis

The screening performances are shown in Table 3 which are presented in term of the number and percentage of cervical cancer and precancer cases detected. Human papilloma virus genotyping as the primary screen with triage of non16/18 HPV-positives using p16/Ki67 dual stain cytology (strategy 2) was the most effective strategy. Almost 90% of cervical cancer cases could be detected by this strategy. Pooled high-risk HPV primary with reflex dual stain cytology was nearly as effective (strategy 3). Relative to Pooled high-risk HPV testing with reflex LBC (strategy 1), the use of dual staining p16/Ki67 test for triage of HPV-positive women did not only increase but also improves the QALY. Human papilloma virus genotyping test with reflex dual stain strategy (strategy 2) decreased the total lifetime cost by US$964 822 and US$4 015 670 when compared to pooled high-risk HPV test alone and pooled high-risk HPV test with reflex dual stain test, respectively. The cost-effectiveness analysis was also presented in the ICER plane (Supplemental Material 1). The result showed the strategies of pooled high-risk HPV test with reflex LBC (strategy 1) were dominated by HPV genotyping with dual stain (strategy 2). Regarding the 2 strategies using reflex dual stain: Primary test with pooled high-risk HPV was not cost effective when compared with primary test with HPV genotyping due to the high ICER value (US$416 912/QALY gain) which revealed more than 3 times of Thailand GPD (Thailand GDP per capita 2017: US$6125.75.^24^ Comparing strategies 3 and 1, pooled high-risk HPV test with reflex LBC was less costly than with dual stain cytology, whereas pooled high-risk HPV test with reflex dual stain provided a more effective strategy at an ICER of US$353.40 per QALY gained. The cost-effectiveness frontier between total cost and QALY gained of each strategy is presented in Supplemental Material 2. Strategy 1 or pooled high-risk HPV testing with reflex LBC was not on the frontier and fell to the right side of the solid line, meaning that it was not an efficient use of resources. Comparing strategy 3 and strategy 2, pooled high-risk HPV test with reflex dual stain cytology was clearly dominated by HPV genotyping with reflex dual staining (same benefit but higher cost).

**Table 3.** Base Case Results of Outcome, Cost, and ICER per QALY Gained.

	HPV Genotyping Test With Reflex Dual Stain (Strategy 2)	Pooled HPV Test With Reflex LBC (Strategy 1)	Pooled HPV Test With Reflex Dual Stain (Strategy 3)
Screening performance (%)			
Cervical cancer detected	88.9	73.5	87.4
Cervical intraepithelial neoplasia 3 detected	85.2	73.3	85.9
Cervical intraepithelial neoplasia 2 detected	79.2	67.7	81.2
Total number of cases detected			
Cervical cancer detected	31 607	35 577	31 765
Cervical intraepithelial neoplasia 3 detected	257 188	240 952	260 658
Cervical intraepithelial neoplasia 2 detected	230 669	200 524	239 577
Average QALY	24.029947	23.98	24.029999
Total cost (USD)	1 326 269 261	1 360 038 064	1 500 586 513
Total cost per person (USD)	167	171	189
Incremental cost per person (USD)	–	4	18
Incremental effectiveness	–	−0.049947433159300	0.0499999999995993
ICER^a^ (USD/ QALY gained)	–	−85^b^	353.405

Abbreviations: HPV, human papilloma virus; ICER; Incremental cost and effectiveness; LBC, liquid-based cytology; QALY, Quality adjust life year.

^a^ The difference in cost divided by the difference in detected case for each strategy compared with the next best strategy.

^b^ Strategies shown cost more but were less effective than the next most expensive strategy and were therefore dominated.

### Sensitivity Analysis

The model sensitivity was measured by the absolute percentage change in ICER induced by decreasing 10% below and increasing 3 times perturbation in the parameters (Supplemental Materials 3 and 4). More significant changes were found in scenarios related to changes in the costs of dual stain cytology test and cancer treatment. However, HPV genotyping test with reflex dual stain still was the optimal strategy.

## Discussion

Evidence suggests that primary HPV testing can be more effective than cytology and additional triage tests are needed to identify women with progressing disease. The triage strategies include HPV genotyping for HPV 16/18, cytology, p16/Ki67 dual stain cytology, host methylation, and viral methylation testing.^25^ It is still not clear what the best strategy for triage of screen-positive women is. To find an optimal integrated screening and triage strategy for Thai women, we conducted the cost-effectiveness analysis study and reported the primary high-risk HPV testing with reflex LBC was preferred over primary liquid base cytology as it was more effective and less costly.^12^ We then applied the HPV model by using p16/Ki67 dual stain cytology as triage for high-risk HPV-positive women (non16/18 HPV positive) and compared with primary cytology. The result showed the good performance of p16/ki67 dual stain cytology and reported that HPV genotyping with reflex dual stain was more costly but more effective than cytology method with an ICER was 1395 per QALY.^14^

From this cost-effectiveness analysis, it can be concluded that HPV primary plus genotyping with reflex dual stain cytology every 5 years for Thai women at age of 30 to 65 was the most effective strategy according to a cost-effectiveness threshold based on per capita GDP. Comparing to pooled high-risk HPV test with reflex LBC, the higher cost of diagnostics test was compensated by higher screening performance and lower cost of treatment. Wright et al assessed the performance of dual stain cytology compared with cytology for triaging HPV-positive women undergoing primary HPV screening. The result showed sensitivity, positive predictive value, and negative predictive value were higher for dual staining than cytology, in a similar way to our result.^16^ Uijterwaal et al reported dual stain cytology had a sensitivity of more than 70% for cervical intraepithelial neoplasia 2/3 and the cumulative 5-year cervical intraepithelial neoplasia 3 risk was significantly reduced in dual stain negative women.^11^ It is therefore suitable for triaging to colposcopy. From our study, the use of dual staining in either strategy 2 or 3 increased the number of precancerous cases detected which resulted in decreased cancer cases detected. One prospective study in HPV-positive women revealed that the dual stain cytology had similar sensitivity and higher specificity compared with cytology for detection cervical intraepithelial neoplasia 2/3 cases.^9^ Until now, there are no data that how long dual stain negative women remain at low risk of precancerous. Women with positive HPV and negative dual stain in our model were retested with HPV testing, while women with positive HPV and negative cytology were returned to routine screening. If the women with positive HPV and negative dual staining were returned to routine safety, the total cost of strategies 2 and 3 would decrease and these 2 strategies would be more cost-effective than cytology triaging.

Comparing between primary pooled high-risk HPV primary testing with dual stain cytology (strategy 3) and primary HPV genotyping with dual stain cytology (strategy 2), the clinical outcomes were comparable but strategy 3 was more costly. Both strategies included dual stain cytology which was one of the most expensive diagnostic tests. A larger number of women in strategy 3 were sent for dual stain cytology while HPV 16/18-positive women in strategy 2 were referred directly to colposcopy. This may cause strategy 3 to be the most expensive strategy. One-way sensitivity analysis also confirmed this reason, if the cost of dual staining increased 3 times, the ICER between strategy 3 versus 1 and 3 versus 2 would increase to 114% and 23.97%, respectively. The cost of cancer treatment was another factor that affected the ICER result, the ICER between strategies 3 versus 2 increased almost twice when cancer treatment cost increased 3 times. In the first screening round, the total cost in strategy 2 may be slightly higher than strategy 3 because more cancer cases were detected and treated. However, the number of women would be reduced in the next screening round and the detection of cancer cases would be lower. This results in decreasing the cost of cancer treatment in long term for strategy 2.

To the best of our knowledge, this is the first economic study to evaluate an optimal triage strategy for HPV-positive women in Thailand. Our analysis included both direct costs of screening and treatment, and we presented the screening performance, a number of cases detected, QALY and screening budget of each strategy. However, there were some limitations of our study. First, several clinical input parameters of the model were derived from Western literature and the percentage of cervical intraepithelial neoplasia cases received treatment were based on the expert’s opinion. Second, since there were no Thai data available, we did not use the age-specific progression rates for HPV acquisition, clearance, progression from HPV infection to cervical intraepithelial neoplasia 2/3 and cancer. Third, our analysis calculated the cost from Thai Government Hospital which limited applicability to health-care settings in other countries. We tried to minimize this limitation by using the asymmetrical distribution of the cost to right-hand tail in sensitivity analysis because the costs we used were lower compared to the average cost of overall hospital in Thailand. To clarify the most suitable strategy for implementation of cervical cancer screening in Thailand, further studies are necessary. The clinical parameters should be based on the updated data from several regions of Thailand. Indirect cost such as cost of day loss should be incorporated to estimate the total cost of illness based on social perspective. Finally, increasing HPV vaccination coverage will lead to lower prevalence of HPV16/18 infection or higher prevalence of other high-risk HPV infection, which will further reduce the efficacy of strategy 2.

In summary, the findings from this study emphasizes the importance of dual stain cytology as a triage test for high risk HPV-positive women. The strategy of primary HPV genotyping test with dual stain cytology every 5 years interval was the most cost-effective screening method and should be considered for implementation in practice and for future guidelines.

## Supplemental Material

Supplementary - Cost-Effectiveness of Primary HPV Screening Strategies and Triage With Cytology or Dual Stain for Cervical Cancer Click here for additional data file. Supplementary for Cost-Effectiveness of Primary HPV Screening Strategies and Triage With Cytology or Dual Stain for Cervical Cancer by Tanitra Tantitamit, Nipon Khemapech, Piyalamporn Havanond and Wichai Termrungruanglert in Cancer Control

## References

[1] World Health Organization. Cervical cancer. 2018 https://www.who.int/cancer/prevention/diagnosis-screening/cervical-cancer/en/. Accessed January 1, 2019.

[2] Gakidou E Nordhagen S Obermeyer Z Coverage of cervical cancer screening in 57 countries: low average levels and large inequalities. PLoS Med. 2008;5(6):e132. 18563963 10.1371/journal.pmed.0050132 PMC2429949

[3] Mukem S Meng Q Sriplung H, et al. Low coverage and disparities of breast and cervical cancer screening in Thai women: analysis of national representative household surveys. Asian Pac J Cancer Prev. 2015;16(18):8541–8551. 26745114 10.7314/apjcp.2015.16.18.8541

[4] Sankaranarayanan R Budukh AM Rajkumar R Effective screening programmes for cervical cancer in low- and middle-income developing countries. Bull World Health Organ. 2001;79(10):954–962. 11693978 PMC2566667

[5] Yothasamut J Putchong C Sirisamutr T, et al. Scaling up cervical cancer screening in the midst of human papillomavirus vaccination advocacy in Thailand. BMC Health Services Research. 2010;10(1):S5. 20594371 10.1186/1472-6963-10-S1-S5 PMC2895749

[6] Ngugi CW Schmidt D Wanyoro K, et al. P16(INK4a)/Ki-67 dual stain cytology for cervical cancer screening in Thika district, Kenya. Infect Agent Cancer. 2015;10:25. 26265934 10.1186/s13027-015-0020-2 PMC4531480

[7] Wright TC Jr Behrens CM Ranger-Moore J, et al. Triaging HPV-positive women with p16/Ki-67 dual-stained cytology: results from a sub-study nested into the ATHENA trial. Gynecol Oncol. 2017;144(1):51–56. 28094038 10.1016/j.ygyno.2016.10.031

[8] Ikenberg H Bergeron C Schmidt D, et al. Screening for cervical cancer precursors with p16/Ki-67 dual-stained cytology: results of the PALMS study. J Natl Cancer Inst. 2013;105(20):1550–1557. 24096620 10.1093/jnci/djt235 PMC3814411

[9] Wentzensen N Fetterman B Castle PE, et al. p16/Ki-67 Dual stain cytology for detection of cervical precancer in HPV-positive women. J Natl Cancer Inst*.* 2015;107(12):djv257. 26376685 10.1093/jnci/djv257 PMC4675094

[10] Wright TC Jr Stoler MH Sharma A, et al. Evaluation of HPV-16 and HPV-18 genotyping for the triage of women with high-risk HPV+ cytology-negative results. Am J Clin Pathol. 2011;136(4):578–586. 21917680 10.1309/AJCPTUS5EXAS6DKZ

[11] Uijterwaal MH Polman NJ Witte BI, et al. Triaging HPV-positive women with normal cytology by p16/Ki-67 dual-stained cytology testing: baseline and longitudinal data. Int J Cancer. 2015;136(10):2361–2368. 25345358 10.1002/ijc.29290

[12] Termrungruanglert W Khemapech N Tantitamit T, et al. Cost-effectiveness analysis study of HPV testing as a primary cervical cancer screening in Thailand. Gynecol Oncol Rep. 2017;22(C):58–63. 29034308 10.1016/j.gore.2017.09.007 PMC5633754

[13] Tjalma WAA. Diagnostic performance of dual-staining cytology for cervical cancer screening: a systematic literature review. Eu J Obstet Gynecol Reprod Reprod Biol 2017;210(suppl C):275–280. 10.1016/j.ejogrb.2017.01.009 28086168

[14] Termrungruanglert W Khemapech N Tantitamit T, et al. Cost effectiveness analysis of HPV primary screening and dual stain cytology triage compared with cervical cytology. J Gynecol Oncol. 2019;30(2):e17. 30740950 10.3802/jgo.2019.30.e17 PMC6393632

[15] Ministry of Public Health. Cervical cancer screening rate in women aged 30-60 years. 2018 https://hdcservice.moph.go.th/hdc/reports/report.php?source=pformated/format1.php&cat_id=6966b0664b89805a484d7ac96c6edc48&id=4eab25b045dc0a9453d85c98dc2fdef0. Accessed June 20, 2018.

[16] National Statistical Office (NSO) UNCsFU, Ministry of Public Health (MOPH), National Health Security Office (NHSO), Thai Health Promotion Foundation (THPF), International Health Policy Program (IHPP). Thailand multiple indicator cluster survey. 2012 http://web.nso.go.th/en/survey/monitoring/data/monitoring_full_report_2012.pdf. Accessed January 20, 2019.

[17] World Bank Group. Death rate, crude (per 1,000 people). 2018 http://data.worldbank.org/indicator/SP.DYN.CDRT.IN. Accessed June 20, 2018.

[18] Friedlander M Grogan M Guidelines for the treatment of recurrent and metastatic cervical cancer. Oncologist. 2002;7(4):342–347. 12185296

[19] Mandelblatt JS Lawrence WF Womack SM, et al. Benefits and costs of using HPV testing to screen for cervical cancer. JAMA. 2002;287(18):2372–2381. 11988058 10.1001/jama.287.18.2372

[20] Prasatkun P Rakchayaban U Estimated Generation Life Tables for Thailand of Five-Year Birth Cohorts: 1900-2000. Nakhon Pathom, Thailand: Institute for Population and Social Research (IPSR), Mahidol University; 2002; pp. 2002.

[21] Termrungruanglert W Havanond P Khemapech N, et al. Cost and effectiveness evaluation of prophylactic HPV vaccine in developing countries. Value Health. 2012;15(1 suppl):S29–S34. 22265063 10.1016/j.jval.2011.11.007

[22] Ogilvie GS van Niekerk D Krajden M, et al. Effect of screening with primary cervical HPV testing vs cytology testing on high-grade cervical intraepithelial neoplasia at 48 months: the HPV FOCAL randomized clinical trial. JAMA 2018;320(1):43–52. 29971397 10.1001/jama.2018.7464 PMC6583046

[23] World Health Organization. Macroeconomic and Health: Investing in Health for Economic Development: Report for the Commission on Macroeconomics and Health. Geneva, Switzerland: World Health Organization; 2001.

[24] Trading Economics. Thailand GDP. 2019 https://tradingeconomics.com/thailand/gdp. Accessed January 20, 2019.

[25] Wentzensen N Schiffman M Palmer T, et al. Triage of HPV positive women in cervical cancer screening. J Clin Virol 2016;76(suppl 1):S49–S55. 26643050 10.1016/j.jcv.2015.11.015 PMC4789103

[26] Wright TC Jr Stoler MH Behrens CM Apple R Derion T Wright TL The ATHENA human papillomavirus study: design, methods, and baseline results. Am J Obstet Genecol 2012;206(1):46 e1-e11. 10.1016/j.ajog.2011.07.024 21944226

[27] Cox JT Castle PE Behrens CM, et al. Comparison of cervical cancer screening strategies incorporating different combinations of cytology, HPV testing, and genotyping for HPV 16/18: results from the ATHENA HPV study. Am J Obstet Genecol. 2013;208(3):184 e1-e11. 10.1016/j.ajog.2012.11.020 23174289

[28] Schneider A Hoyer H Lotz B, et al. Screening for high-grade cervical intra-epithelial neoplasia and cancer by testing for high-risk HPV, routine cytology or colposcopy. Int J Cancer. 2000;89(6):529–534. 11102899

[29] Petry KU Menton S Menton M, et al. Inclusion of HPV testing in routine cervical cancer screening for women above 29 years in Germany: results for 8466 patients. Br J Cancer. 2003;88(10):1570–1577. 12771924 10.1038/sj.bjc.6600918 PMC2377109

[30] Klug SJ Hukelmann M Hollwitz B, et al. Prevalence of human papillomavirus types in women screened by cytology in Germany. J Med Virol. 2007;79(5):616–625. 17385693 10.1002/jmv.20863

[31] Luyten A Scherbring S Reinecke-Luthge A, et al. Risk-adapted primary HPV cervical cancer screening project in Wolfsburg, Germany—experience over 3 years. J Clin Virol 2009;46(suppl 3):S5–S10. 20129072 10.1016/S1386-6532(09)70294-X

[32] World Bank Group. Death rate, crude (per 1,000 people).2014 http://data.worldbank.org/indicator/SP.DYN.CDRT.IN. Accessed June 20, 2018.

[33] Cuzick J Myers O Hunt WC, et al. Human papillomavirus testing 2007-2012: co-testing and triage utilization and impact on subsequent clinical management. Int J Cancer. 2015;136(12):2854–2863. 25447979 10.1002/ijc.29337 PMC4737644

[34] Kataja V Syrjanen K Mantyjarvi R, et al. Prospective follow-up of cervical HPV infections: life table analysis of histopathological, cytological and colposcopic data. Eur J Epidemiol. 1989;5(1):1–7. 2540024 10.1007/BF00145037

[35] Holowaty P Miller AB Rohan T, et al. Natural history of dysplasia of the uterine cervix. J Natl Cancer Inst. 1999;91(3):252–258. 10037103 10.1093/jnci/91.3.252

[36] Khan MJ Castle PE Lorincz AT, et al. The elevated 10-year risk of cervical precancer and cancer in women with human papillomavirus (HPV) type 16 or 18 and the possible utility of type-specific HPV testing in clinical practice. J Natl Cancer Inst 2005;97(14):1072–1079. 16030305 10.1093/jnci/dji187

[37] Matsumoto K Yasugi T Oki A, et al. IgG antibodies to HPV16, 52, 58 and 6 L1-capsids and spontaneous regression of cervical intraepithelial neoplasia. Cancer Lett 2006;231(2):309–313. 16399232 10.1016/j.canlet.2005.02.023

[38] Insinga RP Dasbach EJ Elbasha EH, et al. Progression and regression of incident cervical HPV 6, 11, 16 and 18 infections in young women. Infect Agent Cancer. 2007;2(1):15. 17626624 10.1186/1750-9378-2-15 PMC2034372

[39] Insinga RP Perez G Wheeler CM, et al. Incident cervical HPV infections in young women: transition probabilities for CIN and infection clearance. Cancer Epidemiol Biomarkers Prev. 2011;20(2):287–296. 21300618 10.1158/1055-9965.EPI-10-0791

[40] Insinga RP Dasbach EJ Elbasha EH Epidemiologic natural history and clinical management of human papillomavirus (HPV) disease: a critical and systematic review of the literature in the development of an HPV dynamic transmission model. BMC Infect Dis. 2009;9(1):119. 19640281 10.1186/1471-2334-9-119 PMC2728100

[41] Kulasingam SL Benard S Barnabas RV, et al. Adding a quadrivalent human papillomavirus vaccine to the UK cervical cancer screening programme: a cost-effectiveness analysis. Cost Eff Resour Alloc. 2008;6(1):4. 18279515 10.1186/1478-7547-6-4 PMC2290741

[42] Chen T Jansen L Gondos A, et al. Survival of cervical cancer patients in Germany in the early 21st century: a period analysis by age, histology, and stage. Acta Oncol. 2012;51(7):915–921. 22928692 10.3109/0284186X.2012.708105

