# Supplementary material for: Cost-Effectiveness of Primary HPV Screening Strategies and Triage With Cytology or Dual Stain for Cervical Cancer
Source: Cancer Control. 2020 May 6;27(1):1073274820922540. doi: 10.1177/1073274820922540 (PMC7218320; doi:10.1177/1073274820922540)
Supplement: Supplementary - Cost-Effectiveness of Primary HPV Screening Strategies and Triage With Cytology or Dual Stain for Cervical Cancer [file Supplementary.pdf]

Supplementary 1: The ICER plane of difference strategies

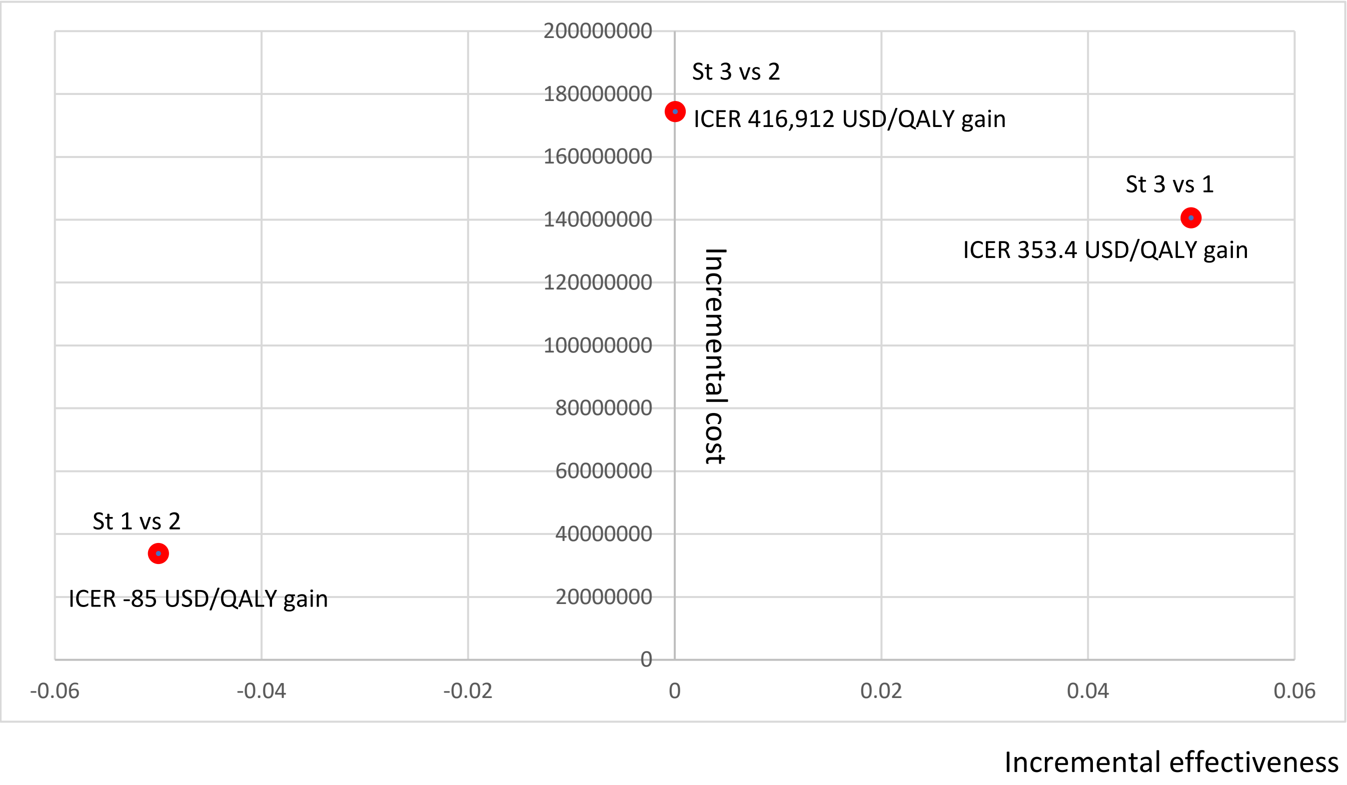

ICER – Incremental cost- effectiveness ratio, St1 – Pooled HPV test, St2 – HPV genotyping test with reflex dual stain, St3 – Pooled HPV test with reflex dual stain

Supplementary 2: Cost-effectiveness analysis compares the strategies based on a CE frontier.

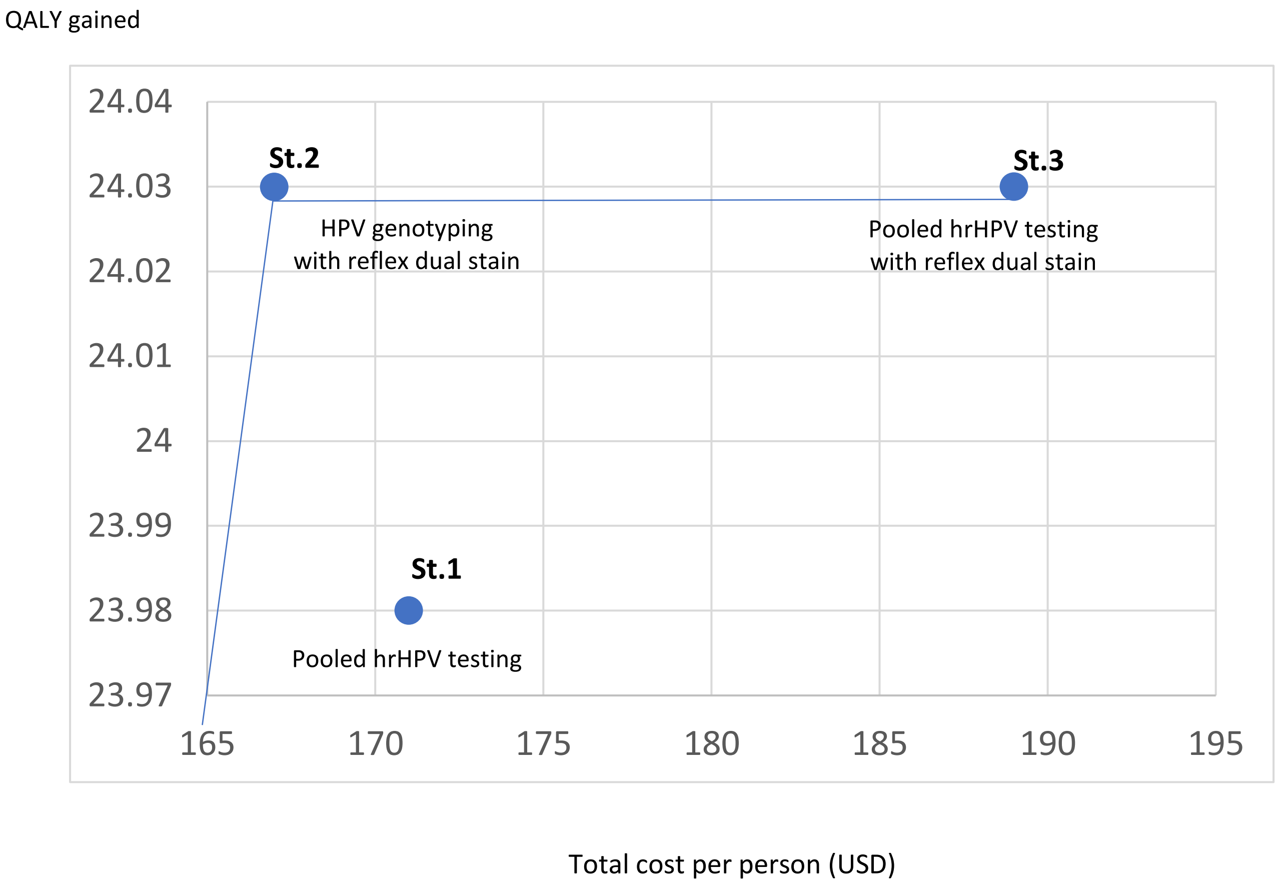

Supplementary 3: Tornado Diagram

5a) ICER Strategy 2 vs 1

(HPV genotyping test with reflex dual stain vs Pooled HPV testing)

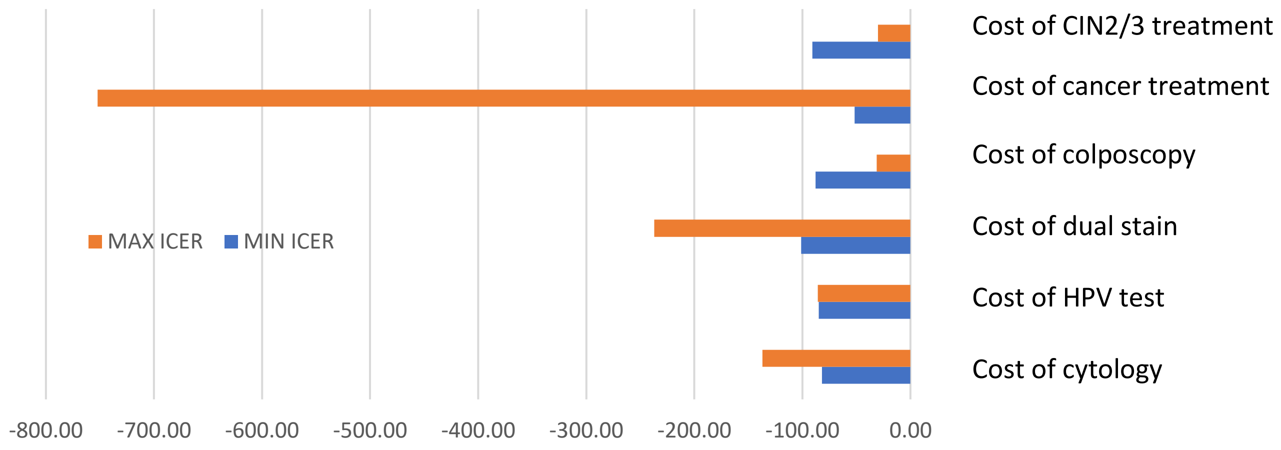

5b) ICER Strategy 3 vs 1

(Pooled HPV test with reflex dual stain vs Pooled HPV test)

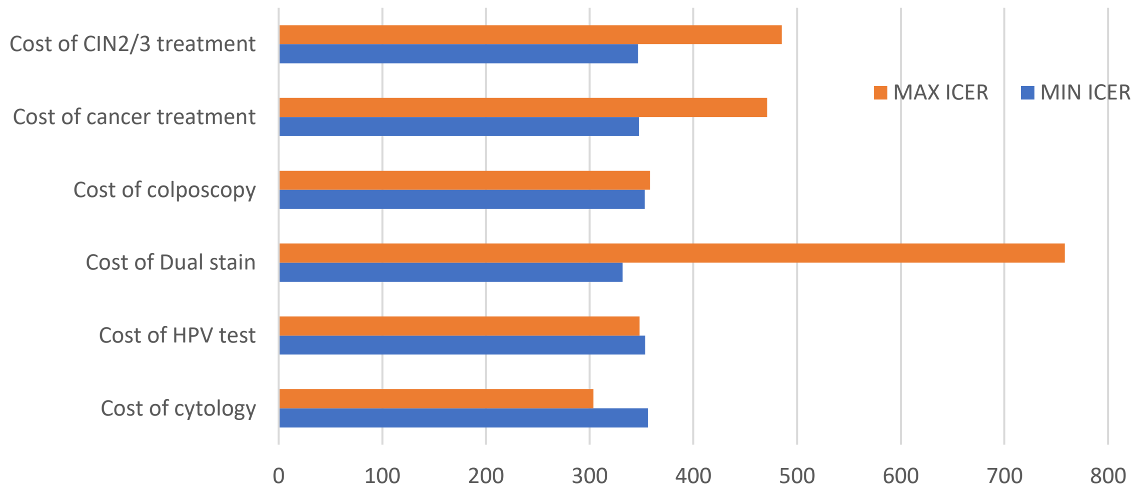

5c) ICER Strategy 3 vs 2

(Pooled HPV test with reflex dual stain vs HPV genotyping test with reflex dual stain)

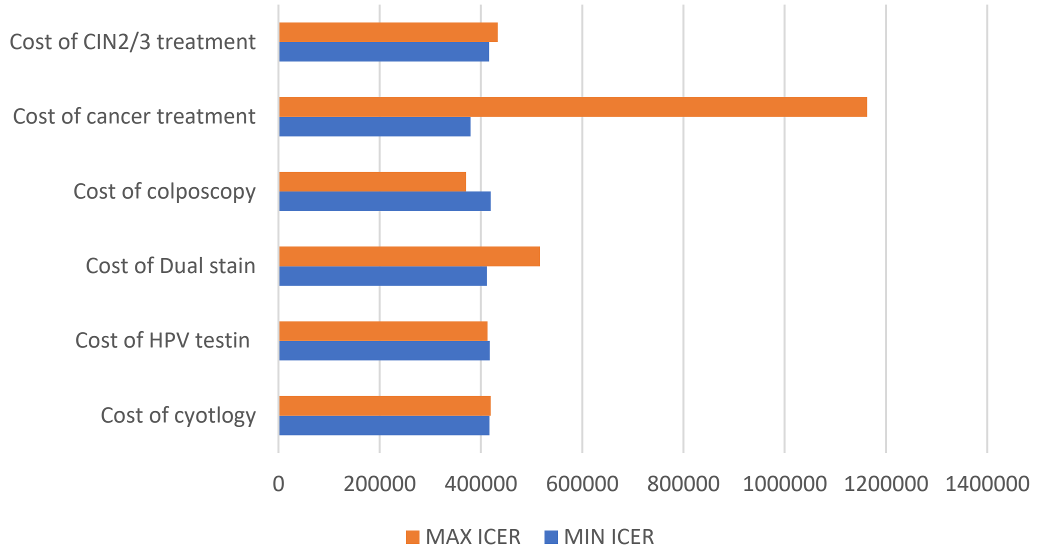

Supplementary 4: One-way sensitivity analysis

Key Parameters	Base case	Value for sensitivity analysis		ICER range (compared with base case ICER)		
				St1 vs S2	St3 vs St1	St3 vs St2
Cost of Cytology	$5.30	Low value	$4.77	-82 (3.5%)	356.22 (0.79%)	416,775.23 (-0.03%)
		High value	$15.90	-137 (-61.17%)	303.56 (-14.1%)	419,337.83 (0.58)
Cost of HPV	$17.00	Low value	$15.30	-84.95 (0.059%)	353.66 (0.072%)	417,124.71 (0.05%)
		High value	$51.00	-85.84 (-0.98%)	348.1 (1.5%)	412,688.71 (-1.01)
Cost of dual stained Cytology test	$35.00	Low value	$31.50	-101.09 (-18.9%)	331.81 (6.1%)	411,655.29 (-1.26)
		High value	$105.00	-236.97 (-178.78%)	758.37 (114.5%)	516,856.9 (23.97)
Cost of colposcopy	$21.42	Low value	$19.27	-87.70 (-3.07%)	353.16 (-0.069%)	419,246.9 (0.56%)
		High value	$64.26	-31.10 (63.4%)	358.2 (1.33%)	370,398.28 (-11.16%)
Cancer Treatment Cost	$7,403.00	Low value	$6,662.70	-51.63 (39.25%)	347.51 (1.6%)	379,566.34 (-8.96)
		High value	$22,209.00	-752.29 (-785.03%)	471.34 (33.3%	1,163,130.32 (178.99)
Treatment Cost for CIN2/3	$1,292.00	Low value	$1,162.80	-90.78 (-6.79%)	346.76 (1.8%)	416,095.62 (-0.2%)
		High value	$3,876.00	-30.02 (64.68%)	485.37 (37.3%)	433,149.63 (3.89%)

(Thailand GDP per capita 2017: 6,125.75 USD = 200,787,7 THB
